# Investigation of Baseline Iron Levels in Australian Chickpea and Evaluation of a Transgenic Biofortification Approach

**DOI:** 10.3389/fpls.2018.00788

**Published:** 2018-06-14

**Authors:** Grace Z. H. Tan, Sudipta S. Das Bhowmik, Thi M. L. Hoang, Mohammad R. Karbaschi, Hao Long, Alam Cheng, Julien P. Bonneau, Jesse T. Beasley, Alexander A. T. Johnson, Brett Williams, Sagadevan G. Mundree

**Affiliations:** ^1^Centre for Tropical Crops and Biocommodities, Queensland University of Technology, Brisbane, QLD, Australia; ^2^School of Biosciences, The University of Melbourne, Melbourne, VIC, Australia

**Keywords:** pulse biofortification, iron, genetic modification, nicotianamine synthase, soybean ferritin, crop improvement, chickpea (*Cicer arietinum* L.)

## Abstract

Iron deficiency currently affects over two billion people worldwide despite significant advances in technology and society aimed at mitigating this global health problem. Biofortification of food staples with iron (Fe) represents a sustainable approach for alleviating human Fe deficiency in developing countries, however, biofortification efforts have focused extensively on cereal staples while pulses have been largely overlooked. In this study we describe a genetic engineering (GE) approach to biofortify the pulse crop, chickpea (*Cicer arietinum* L.), with Fe using a combination of the chickpea nicotianamine synthase 2 (*CaNAS2*) and soybean (*Glycine max*) ferritin (*GmFER*) genes which function in Fe transport and storage, respectively. This study consists of three main components: (1) the establishment for baseline Fe concentration of existing germplam, (2) the isolation and study of expression pattern of the novel *CaNAS2* gene, and (3) the generation of GE chickpea overexpressing the *CaNAS2* and *GmFER* genes. Seed of six commercial chickpea cultivars was collected from four different field locations in Australia and assessed for seed Fe concentration. The results revealed little difference between the cultivars assessed, and that chickpea seed Fe was negatively affected where soil Fe bioavailability is low. The desi cultivar HatTrick was then selected for further study. From it, the *CaNAS2* gene was cloned and its expression in different tissues examined. The gene was found to be expressed in multiple vegetative tissues under Fe-sufficient conditions, suggesting that it may play a housekeeping role in systemic translocation of Fe. Two GE chickpea events were then generated and the overexpression of the *CaNAS2* and *GmFER* transgenes confirmed. Analysis of nicotianamine (NA) and Fe levels in the GE seeds revealed that NA was nearly doubled compared to the null control while Fe concentration was not changed. Increased NA content in chickpea seed is likely to translate into increased Fe bioavailability and may thus overcome the effect of the bioavailability inhibitors found in pulses; however, further study is required to confirm this. This is the first known example of GE Fe biofortified chickpea; information gleaned from this study can feed into future pulse biofortification work to help alleviate global Fe deficiency.

## Introduction

Iron (Fe) deficiency has long been recognized as one of the most common micronutrient deficiencies in the world. Afflicting both developing and developed nations, it is the cause of more than 60% of global anemia cases (WHO, 2008; Alvarez-Uria et al., 2014). To combat this problem several strategies have been developed such as dietary diversification, supplementation, food fortification, and crop development. Amongst these, the development of crops with increased Fe concentrations and/or bioavailability (also known as “biofortification”) has garnered great interest due to its sustainability, cost-effectiveness, and accessibility of products to vulnerable populations (Nestel et al., 2006).

Biofortification can be achieved through breeding or genetic engineering (GE), and this has been performed in various crop species. The focus thus far, however, has mostly been on starchy staples like as cereals (e.g., rice, wheat, pearl millet) and root crops (e.g., potato, cassava) (HarvestPlus, 2015). Naturally, it is in these species that the greatest advances have been made. For instance, more than three-fold increase in Fe concentration has been reported in biofortified pearl millet and its effectiveness in combating Fe deficiency anemia has been verified via feeding trials (Cercamondi et al., 2013; Finkelstein et al., 2015).

Aside from the aforementioned staples, recent years have seen growing interest in pulses as targets for Fe biofortification. Pulses are defined as leguminous crops harvested solely for dry grain (FAO, 1994), and most serve as important secondary staples, particularly with their high protein content (Iqbal et al., 2006); it is this latter feature that also complements the existing biofortification work in cereals. The pulse biofortification effort is relatively young compared to cereals but there has been considerable progress, notably in the common bean. Several biofortified varieties have been generated from the HarvestPlus breeding programs, with up to 94% enhancement in seed Fe concentration achieved (Katsvairo, 2015). The work has also progressed to feeding trials which have yielded promising results (Cercamondi et al., 2013; Kodkany et al., 2013; Finkelstein et al., 2015). This success has paved the way for advances in other pulses. Studies on Fe accumulation traits have been performed on cowpea (Fernandes Santos and Boiteux, 2015), chickpea (Diapari et al., 2014), pea and lentil (Ray et al., 2014), and test trials are currently underway for some of them (HarvestPlus, 2016).

Thus far, most, if not all, of this work has been focused on breeding while GE remains unexplored. As such, there are no established GE strategies for pulses, though some lessons can be drawn from the work in cereals. One of the most successful examples to date is the GE Fe biofortified rice (*Orzya sativa*), in which seed Fe concentration was increased by 7.5-fold with no yield penalty (Trijatmiko et al., 2016). The strategy targeted the three core processes of Fe metabolism—uptake, translocation, and storage—through constitutive overexpression of the rice Nicotianamine Synthase 2 gene (*OsNAS2*) gene and seed-specific expression of the soybean (*Glycine max*) Ferritin (*GmFER*) gene.

NAS catalyzes the biosynthesis of nicotianamine (NA), a non-proteogenic chelator of divalent transition metals that facilitates translocation of said metals in plants (Scholz et al., 1992). In graminaceous species, it is also a precursor for the mugeneic acid (MAs) family of phytosiderophores which contribute to both Fe uptake from soil and *in planta* translocation (Higuchi et al., 1999). When constitutively overexpressed in rice cv. Nipponbare, the *OsNAS2* gene caused a four-fold increase in grain Fe concentration (Johnson et al., 2011).

Ferritin (FER), on the other hand, is an Fe storage protein that allows for safe sequestration of Fe in a soluble and bioavailable form. When overexpressed in the seed, *GmFER* has been demonstrated to increase seed Fe concentration by up to threefold in several plant species (Goto et al., 1999). Excessive expression, however, may lead to disproportionate sink strength, resulting in altered sequestration of Fe in source tissues and the development of Fe deficiency symptoms (Van Wuytswinkel et al., 1999; Qu et al., 2005; Masuda et al., 2013b). This problem can be rectified by increasing Fe uptake and translocation capacities, such as through the co-expression with NAS (Masuda et al., 2013a). In this case, a synergistic effect was also achieved, producing greater enhancement in seed Fe concentration (Wirth et al., 2009; Trijatmiko et al., 2016).

Whether this strategy will have a similar effect on Fe concentration when applied to a pulse crop is uncertain. However a major advantage is its potential effect on Fe bioavailability. Both NAS and GmFER have been linked to increased Fe biovailability (Davila-Hicks et al., 2004; Lönnerdal et al., 2006; Zheng et al., 2010), a feature not usually accorded to other commonly used Fe metabolism genes. This is particularly relevant to pulse biofortification given the inherently high levels of antinutrients like phytic acid which inhibit Fe absorption in the gut (Sandberg et al., 1989; Hemalatha et al., 2007; Petry et al., 2014).

For this study, target species is chickpea (*Cicer arietinum*). The second most important pulse crop in the world with an annual production exceeding 14.2 million tons (FAO, 2016). The bulk of the chickpea crop is currently grown and consumed in India where human Fe deficiency is prevalent, however, continued population growth is likely to result in increased demand for chickpea in Africa and other parts of Asia (Rao et al., 2010; Akibode and Maredia, 2012). Fe concentrations in chickpea has been found to range from 3 to 14.3 ppm (Wood and Grusak, 2007), though due to the presence of naturally occurring inhibitors, only a small fraction is bioavailable (Hemalatha et al., 2007). Both iron concentration and bioavailability is subject to genotype and environmental effects, and to date, detailed studies of such effects are limited to populations in India (Upadhyaya et al., 2016) and Canada (Diapari et al., 2014). No such information is available for Australian populations, and part of this study would therefore serve to partially fill in this gap.

Overall, the main aim of this study was to biofortify chickpea by GE to overexpress NAS and GmFER. This body of work consisted of three parts: (1) assessing the macro- and micro-elemental composition of six modern Australian chickpea cultivars and identifying a suitable cultivar for Fe biofortification research, (2) cloning and expression analysis of an endogenous chickpea NAS gene termed *CaNAS2*, and (3) constitutive overexpression of the *CaNAS2* gene and constitutive expression of soybean *GmFER* in chickpea as a novel GE approach to produce Fe biofortified chickpea.

## Materials and methods

### Plant material

For the elemental composition analysis of commercial chickpea, three kabuli cultivars (Genesis090™, Kalkee™, and PBA Monarch) and three desi cultivars (PBA Boundary, CICA0912, and PBA HatTrick) were used. Seed samples were obtained from field trials at four locations within Queensland—Billa Billa, Warra, Roma, and Kingaroy—and from the seed company Grainland in Moree, New South Wales. All seeds were produced during the 2014 winter growing season. Information on the cultivation sites and conditions during the growing period, where available, is listed in Supplementary Table 1. The soil types of the field locations were provided by Dr Yash Chauhan from the Agricultural Production Systems sIMulator (APSIM) database.

For the gene expression analyses and chickpea transformation, PBA HatTrick seeds were purchased from the seed company Grainland in Moree, New South Wales.

### Plant growth conditions

All *in vitro* cultivation was performed in growth cabinets set at 24 ± 1°C, under fluorescent lights with a 16 h light/ 8 h dark cycle.

For glasshouse cultivation, temperatures were maintained at 21 ± 1°C and 61 ± 1.5% relative humidity. Natural lighting was used except during dusk, when artificial lighting was then turned on to complete a 16 h light/ 8 h dark cycle. An average natural light intensity of around 450 ± 1 μmoles s-1 m-2 of cloudy and sunny day prevailed during the growth period. Seeds were first germinated in Plugger's potting mix, before transplanting to 400 × 250 mm pots containing a 1:1 mixture of University of California (UC) mix and Searles® Premium potting mix. The recipe for the UC mix consists of 80 kg sand, 120 kg peat, and 100 kg sand, peat, and gravel, supplemented with 400 g blood and bone, 100 g Micromax micronutrients, 40 g KSO_4_, 40 g KNO_3_, 400 g superphosphate, 300 g hydrated lime, and 1,200 g dolomite.

Plant were watered with 100 mL every 2 days via an automated watering system. After ~3 months, when at least 80% of the pods have filled, watering was ceased in preparation for harvesting. Harvesting was done approximately 3 weeks thereafter, or when the plants have completely dried. All seeds were de-husked by hand and stored in paper envelopes at 4°C until planting or analysis.

### Elemental analysis

All samples were cleaned, freeze-dried, and milled prior to analysis. A minimum of three biological replicates were used per transgenic event.

For leaf tissue, milled samples were pressed into 5 mm diameter pellets and analyzed via LA-ICP-MS (laser ablation inductively-coupled mass spectroscopy) using an Agilent 8,800 Inductively Coupled Plasma Mass Spectrometer attached with an ESI 193 nm Excimer Laser. The laser was set at a pulse width of 4 ns, spot size of 85 microns, and scan speed of 10 microns/s. At least three lines scans were used for each sample as technical replicates.

For whole seed analysis, acid digestion was performed on milled samples. Briefly, 2 mL HNO_3_ and 0.5 mL H_2_O_2_ were added to 200–300 mg of milled sample, vortexed, and allowed to stand overnight at room temperature. Following digestion, the tubes were shaken at 200 rpm for 20 min, incubated at 80°C for 30 min, then 125°C for 2 h. Upon cooling to room temperature, the volume was made to 25 mL using MilliQ water and the samples agitated at 300 rpm for 5 min. Undissolved material (e.g., silicates) was settled for 60 min. The settled extract was then filtered and analyzed via ICP-OES (inductively coupled plasma optical emission spectroscopy) using a Perkin Elmer Optima 8300 DV Inductively Coupled Plasma Optical Emission Spectrometer. Three technical replicates were prepared per sample.

For analysis of trace element distribution in the seed, 100 seeds were imbibed in MilliQ water for 20 h. The seeds were then separated into the seed coat, cotyledons, and radicle. To measure approximate distribution of mass, the weight of the individual parts of 10 seeds were taken. Tissues of the same type were then pooled and processed for analysis like the whole seed. Three technical replicates were prepared per sample.

### Designation and bioinformatics analysis of chickpea NAS2 gene

Four chickpea NAS amino acid sequences (XP_004495658.1, XP_004487761.1, XP_004488704.1 and XP_004494544.1) were retrieved from the NCBI database. The ortholog with highest similarity to the amino acid sequence encoded by the rice *OsNAS2* gene (LOC_Os03g19420) was designated as the *CaNAS2* gene coding for protein XP_004495658.1 (Supplementary Table 2). The three additional NAS genes were named as the following: *CaNAS3* coding for XP_004487761.1 protein, *CaNAS4* coding for XP_004494544.1 protein, and *CaNAS1* for XP_004488704.1 protein respectively. The four NAS amino acid sequences were aligned based on amino acid conservation using the Geneious Pro 5.6.6, as per the settings described by Bonneau et al. (2016) (CLUSTALW—cost matrix BLOSUM62, threshold 1). A blastn using the genomic sequences of the four *CaNAS* genes was performed against *Cicer arietinum* (cv. kabuli, CDC Frontier)—CDS database (https://legumeinfo.org) to identify chromosomal location. Several bioinformatics tools were then used to predict characteristics of the enzyme encoded by CaNAS2: the theoretical isoelectric point (pI) and molecular weight were calculated using the Compute pI/Mw tool on ExPASY (Bjellqvist et al., 1993, 1994; Gasteiger et al., 2005); the hydrophobicity profile of the protein was assessed using ProtScale (Gasteiger et al., 2005) and potential transmembrane sections were identified using TMpred (http://www.ch.embnet.org/software/TMPRED_form.html). A check for motif sequences was conducted using ScanProsite (de Castro et al., 2006) and MOTIF Search *GenomeNet*^1^ Phobius (Käll et al., 2004) and iPSORT (Bannai et al., 2001, 2002) was used to identify potential signaling peptides.

### Phylogenetic analysis of NAS proteins

A progressive pairwise alignment was performed using full length amino acid sequences of 58 NAS proteins (Supplementary Table 3) using default settings of Geneious alignment (global alignment with free end gaps, Blosum62, gap open penalty 12, gap extension penalty 3)—Geneious Pro 8.1.7 software. Once the protein alignment, a phylogenetic analysis which generated an unrooted tree was conducted as described in Beasley et al. (2017).

### RNA extractions and quantitative RT-PCR

Total RNA was isolated from frozen tissues using RNeasy Mini kits (QIAGEN) following the manufacturer's instructions. A 500 ng aliquot of total RNA was treated with RQ1-DNAse (Promega) and the absence of contaminating DNA confirmed via PCR. cDNA was then synthesized using SuperScript™ IV Reverse Transcriptase (ThermoFisher Scientific). The synthesized cDNA was used for qualitative RT-PCR and quantitative RT-PCR.

For qualitative RT-PCR, each reaction comprised of 5 μL of 2X GoTaq green (Promega), 0.25 μL each of 10 μM forward and reverse primers, 0.6 μL of DMSO, and 1 μL of undiluted cDNA as the template. MilliQ water was added to reach a final reaction volume of 10.6 μL. The PCR program used was as such: initial denaturation at 95°C for 3 min, followed by 30 cycles of denaturation at 95°C for 30 s, annealing at 48–60°C (depending on primers) for 30 s, and extension at 72°C. Extension time was set at 1 min per 1 kbp of the final product size. A final extension was done at 72°C for 5 min.

For qPCR, a 1:30 dilution of cDNA was used as template. The latter was performed on a CFX384 Touch™ Real-Time PCR Detection System (BIO-RAD) using the SYBR Green PCR Master Mix kit (Applied Biosystems). A primer concentration of 300 nM was used, and the primer sequences are as listed in Supplementary Table 4. The housekeeping genesEF1α, and GAPDH were included in each run to serve as internal controls; their primer sequences are as published by Garg et al. (2010). All housekeeping genes were confirmed to be stable under the experimental conditions used. Three biological replicates were used, from each of which three technical replicates were prepared. The qPCR program used was as follows: initial denaturation at 95°C for 10 min, followed by 45 cycles of denaturation at 95°C for 10 s, annealing at 60°C for 30 s, and slow ramping of 0.5°C/min from 65 to 90°C for the melt curve.

### Fe deficiency experiment

PBA Hattrick seeds were sterilized and germinated on half strength Murashige and Skooge (MS) media. Seed coats were removed post-germination and the seedlings were acclimatized for 4 days in tap water. Twenty four-week old seedlings of approximately the same size and developmental stage were transferred to a hydroponics system in a growth cabinet, with 10 replicates per set-up. Later, 1 month old plants were treated with full-strength Hoagland solution with or without Fe-EDTA. Each setup contained ~600 mL of Hoagland solution which was topped up every 3 days. The Hoagland solution was replaced with MilliQ water every third top-up to dilute any accumulated salts. Visible chlorosis in the Fe-deprived plants was observed after 4 weeks of treatment and samples were collected 2 weeks thereafter. Three plants of similar conditions and growth stage were selected from each treatment and the following tissue types collected: mature leaf, stem, cotyledon, and root. Senescent leaf and chlorotic leaf were also collected from the Fe-sufficient and Fe-deficient plants respectively. All samples were snap-frozen in liquid nitrogen immediately after collection and stored in −80°C until RNA extraction.

### Cloning of CaNAS2 and construction of NAS-GmFER overexpression vectors

**Figure 1:**
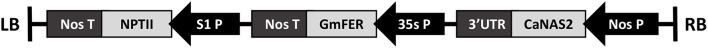
Schematic representation of the T-DNA used for overexpression of CaNAS2 and GmFER in chickpea. LB, left border; Nos T, nopaline synthase terminator; NPTII, neomycin phosphotransferase II gene; S1 P, S1 promoter; GmFER, coding sequence of soybean ferritin H2 gene; 35s P, cauliflower mosaic virus 35s promoter; 3'UTR, cauliflower mosaic virus 3'UTR terminator; CaNAS2, coding sequence of chickpea nicotianamine synthase 2 gene; Nos P, nopaline synthase promoter; RB, right border.

A binary vector using the pOPT-EBX backbone was constructed to constitutively overexpress the *CaNAS2* gene and constitutively express the *GmFER* gene (Figure 1). Included in the T-DNA region was the selectable marker gene neomycin phosphotranferase II (*NPTII*) which confers resistance to the antibiotics geneticin and kanamycin. All cloning primers used are listed in Supplementary Table 5. The *CaNAS2* gene was cloned from chickpea (cv HatTrick) genomic DNA, with primers designed from the predicted sequence in the Genbank database, accession number XM_004495601. Restriction sites were added to the ends via site-directed mutagenesis using high fidelity PCR (Phusion®, NEB). The amplified fragment was cloned into a pGEM®-T Easy, then subcloned in a 5′ to 3′ direction to a pGEM®-T Easy vector with a cassette containing a Nos promoter and CaMV 3′ UTR. Following this the NosP-CaNAS-CaMV 3′ UTR was digested and ligated to a pOpt-EBX-GmFER backbone to form the complete vector. To generate the pOpt-EBX-GmFER backbone, GmFER was cloned from a synthesized fragment (accession no. NM_001250105.2). The amplified fragment was cloned into a pGEM®-T Easy vector, then subcloned into the pOpt-EBX backbone containing a CaMV 35s promoter and Nos terminator.

To ensure integrity and correct orientation of each gene and component, sequence verification was performed after each cloning step in the above process. The final verification was performed on the completed construct, which was then transformed into electrocompetent *Agrobacterium tumefaciens* strains Agl1.

### Generation and molecular characterisation of transgenic chickpea

*Agrobacterium*-mediated transformation of the desi cultivar, PBA Hattrick, followed the protocol developed by Sarmah et al. (2004) with a few modifications. Briefly, half-embryonic axes were prepared from imbibed seeds. Additional injury was inflicted to the cut surface of the radicle using a sterile 26 gauge needle dipped in *Agrobacterium* strain AGL-1 harboring the expression vectors. The explants were immersed in *Agrobacterium* for an hour, followed by co-cultivation in B5 media for 72 h. Following co-cultivation, the explants were transferred to regeneration and selection medium 1 (MS media containing 500 μg/L of BAP, 500 μg/L of kinetin, 50 μg/L of NAA, 200 mg/L of kanamycin and 25 mg/L of meropenem). Shoots obtained in first round of regeneration and selection medium were further selected by subsequent subculturing in the regeneration and selection medium 2 (MS media containing 500 μg/L of BAP, 500 μg/L of kinetin, 200 mg/L of kanamycin and 25 mg/L of meropenem) every 14–21 days. Up to eight rounds of selection were done to obtain putative GE events. Any explants that exhibited proliferative shoot growth during that duration were isolated and considered an individual GE event. Upon reaching an appropriate size, shoots from such multiplying clumps were grafted onto non-GE rootstocks grown on half-strength MS media. Grafts were allowed to set for up to 3 weeks before acclimatization.

Acclimatized plants were screened via PCR of gDNA for the genes of interest. To avoid false negatives caused by potential chimerism, a pooled sample consisting of leaves from every branch was used. Primers used for PCR screening are as listed in Supplementary Table 6. Two PCR-positive T_0_ events were propagated to maturity for two generations to obtain sufficient T_3_ seed for the experiments described in this paper. Null segregants from every generation were also maintained to serve as negative controls.

### NA quantification

Freeze-dried seeds from three different plants of the same transgenic event were pooled and milled to form a bulked flour sample, from which four technical replicates were drawn for NA quantification. Liquid chromatography-mass spectrometry (LC-MS) was used to quantify 9-fluorenylmethoxycarboxyl chloride (FMOC-Cl) derivatized NA on an LC 1290 series coupled to a 6490 series triple quadrupole MS (Agilent Technologies Inc.) using established protocols (Selby-Pham et al., 2017). In short, a combined methanol (100%) and 18 MΩ H_2_O extraction (5 μL) of pulverized chickpea flour (25 mg) was combined with sodium borate buffer (pH = 8, 1 M, 10 μL), EDTA buffer (pH = 8, 50 mM, 10 μL), and fresh FMOC-Cl solution (50 mM, 40 μL). After incubation (60°C, 700 rpm, 15 mins), the solution mixture was quenched via the addition of formic acid (pH = 4, 5%, 8.9 μL). Chromatography was performed using a reverse-phase column (Zorbax Eclipse XDB-C18, HS 2.1 × 100 mm 1.8 Micron, Agilent Technologies Inc.) with aqueous (0.1% v/v FA in dH_2_O) and organic (0.1% v/v FA in acetonitrile) mobile phases.

### Statistical analysis

All statistical analysis was performed on Minitab statistical software (Arend, 2010) using one-way ANOVA. The Tukey HSD test was used in the analysis the different chickpea cultivars, while Dunnett's test was used in the analysis of the GE chickpea.

## Results

### Mineral composition of chickpea cultivars and identification of factors influencing seed Fe concentration

Fe concentrations in chickpea were found to range from 3.36 to 52.0 ppm, with no significant differences between the cultivars, though average values were slightly higher in the kabuli types compared to the desi (Tables 1, 2). The highest values were noted in the kabuli cultivar Genesis090™, while the lowest values were mostly found in PBA HatTrick, though the difference to other desi cultivars was negligible (Table 2). This, in combination with the availability of established transformation protocols for the cultivar, made HatTrick the choice candidate for further work.

**Table 1:** Summary of Fe, Zn, and P concentrations in kabuli and desi cultivars grown at different locations.

		***Kabuli***		***Desi***		***PBA HatTrick***	
	**Location**	**Range**	**Mean**	**Range**	**Mean**	**Range**	**Mean**
Fe	Billa Billa	41.2–46.7	44.2^bc^	39.7–45.3	4.20^c^	39.7–43.3	41.0
	Roma	44.3–52.2	48.7^a^	41.0–49.1	4.45^abc^	41.0–43.0	42.3
	Warra	41.3–55.4	46.3^ab^	42.3–46.5	4.46^bc^	42.3–43.6	43.1
	Kingaroy	n/a	n/a	33.1–42.4	3.63^d^	35.7–42.4	43.1
	NSW	n/a	n/a	n/a	n/a	40.8–437	42.3
Zn	Billa Billa	29.9–33.7	32.3^c^	2.78–3.19	3.02^c^	30.1–31.0	30.6
	Roma	38.6–44.2	41.9^a^	3.85–4.33	4.02^a^	38.5–39.3	30.6
	Warra	31.8–41.2	36.1^b^	3.82–3.96	3.88^ab^	38.4–38.8	38.6
	Kingaroy	n/a	n/a	2.53–3.74	2.96^c^	25.3–26.8	26.0
	NSW	n/a	n/a	n/a	n/a	33.9–36.1	34.9
P	Billa Billa	3,700–4,100	3,890^ab^	3,400–4,100	3,820^abc^	3,400–3,900	3,630
	Roma	2,700–3,500	3,220^d^	3,000–4,000	3,430^cd^	3,000–3,100	3,030
	Warra	3,100–4,400	3,670^bc^	4,000–4,300	4,120^a^	4,100–4,300	4,200
	Kingaroy	n/a	n/a	1,830–2,480	2,040^e^	1,940–2,020	1,990
	NSW	n/a	n/a	n/a	n/a	4,000–4,200	4,080

*All values are expressed as ppm. Data are expressed as mg/100 g and presented as a mean of all cultivars collected from that site. For each cultivar per site, n = > 3. Values with different superscript letters indicate a significant difference at p < 0.05*.

**Table 2:** Concentration of micro-elements in dry chickpea seeds.

	**Fe (ppm)**		**Zn (ppm)**		**Mn (ppm)**		**B (ppm)**		**Cu (ppm)**	
**GENESIS090**										
Billa Billa	^abcd^46.0	(± 0.70)	^fgh^33.3	(± 0.40)	^defgh^29.8	(± 1.40)	^bcdef^10.1	(± 0.10)	^fg^7.40	(± 0.00)
Roma	^a^52.0	(± 0.20)	^a^43.5	(± 0.70)	^fgh^27.3	(± 0.90)	^bcd^10.7	(± 0.50)	^abc^9.90	(± 0.30)
Warra	^ab^50.0	(± 4.60)	^bcd^39.2	(± 2.10)	^cdefg^32.5	(± 1.80)	^b^11.1	(± 1.10)	^abcd^9.50	(± 0.80)
**KALKEE**										
Billa Billa	^cdef^42.2	(± 1.10)	^fghi^32.5	(± 0.40)	^cdefg^32.3	(± 0.90)	^bcd^10.6	(± 0.40)	^efg^7.90	(± 0.10)
Roma	^abc^48.3	(± 0.40)	^ab^42.2	(± 1.20)	^efgh^28.5	(± 1.00)	^bcd^10.5	(± 0.50)	^a^10.1	(± 0.10)
Warra	^bcde^44.2	(± 4.90)	^efg^35.0	(± 3.50)	^bcd^36.8	(± 3.50)	^bcd^10.6	(± 0.40)	^abcde^8.90	(± 0.80)
**MONARCH**										
Billa Billa	^bcde^44.3	(± 0.90)	^ghij^30.9	(± 0.90)	^bcdef^34.2	(± 0.90)	^fghi^8.60	(± 0.60)	^g^6.90	(± 0.20)
Roma	^abcd^45.8	(± 1.70)	^abc^39.8	(± 2.00)	^gh^26.6	(± 1.80)	^bcde^10.4	(± 0.50)	^abcd^9.50	(± 0.50)
Warra	^bcd^44.7	(± 1.00)	^fgh^34.0	(± 1.90)	^abc^38.6	(± 1.90)	^bc^11.0	(± 1.00)	^abcde^9.00	(± 0.60)
**BOUNDARY**										
Billa Billa	^def^41.5	(± 0.80)	^ghi^31.2	(± 1.10)	^bcdef^34.1	(± 4.10)	^defghi^9.10	(± 0.20)	^fg^7.60	(± 0.20)
Roma	^abcd^46.6	(± 2.50)	^ab^41.4	(± 1.60)	^defgh^30.9	(± 0.90)	^bcd^10.5	(± 0.10)	^ab^10.1	(± 0.50)
Warra	^bcd^45.3	(± 0.60)	^bcde^38.5	(± 0.40)	^abc^38.8	(± 2.30)	^bcdefg^9.50	(± 0.30)	^def^8.50	(± 0.30)
Kingaroy	^g^33.6	(± 0.60)	^jk^27.0	(± 0.50)	^ab^41.1	(± 1.10)	^hi^8.40	(± 0.10)	^gh^6.70	(± 0.10)
**CICA0912**										
Billa Billa	^cdef^43.2	(± 1.80)	^ijk^28.9	(± 1.00)	^bcd^36.1	(± 2.10)	^efghi^8.80	(± 0.10)	^efg^7.90	(± 0.20)
Warra	^bcd^45.5	(± 1.10)	^bcd^39.3	(± 0.30)	^bcd^36.6	(± 4.90)	^bc^11.0	(± 1.30)	^abcde^9.00	(± 0.30)
Kingaroy	^fg^37.0	(± 1.50)	^cdef^35.8	(± 1.50)	^ab^40.4	(± 0.50)	^gc^7.80	(± 0.20)	^hi^5.50	(± 0.30)
**HATTRICK**										
Billa Billa	^def^41.4	(± 1.80)	^hij^30.6	(± 0.40)	^ab^41.2	(± 2.90)	^bcdefg^9.60	(± 0.70)	^fg^7.60	(± 1.10)
Roma	^cdef^42.3	(± 1.10)	^bcde^39.0	(± 0.40)	^bcd^36.0	(± 0.60)	^bcdefg^9.70	(± 0.30)	^bcdef^8.8.	(± 0.10)
Warra	^cdef^43.1	(± 0.70)	^bcde^38.6	(± 0.20)	^bcde^35.4	(± 4.80)	^cdefgh^9.40	(± 0.50)	^cdef^8.60	(± 0.10)
Kingaroy	^efg^38.2	(± 3.60)	^k^26.0	(± 0.80)	^a^44.4	(± 2.00)	^i^7.60	(± 0.10)	^i^4.50	(± 0.10)
NSW	^def^42.3	(± 1.20)	^def^34.9	(± 0.90)	^h^25.4	(± 0.70)	^a^14.1	(± 0.20)	^i^4.40	(± 0.30)

*Data is presented as a mean ± SD. All cultivars from had n = 3 except for HatTrick from NSW, which has n = 5. Values sharing the same superscript letters indicate groups that are not significantly different at p < 0.05 when tested with one-way ANOVA, using Tukey's HSD post-hoc test*.

Between locations, similar mineral profiles were observed amongst samples grown in Billa Billa, Roma, and Warra, which had vertosol-type soils. In contrast, samples obtained from Kingaroy contained less Fe. Also, unique to this locality was a high Mn concentrationto Fe ratio, which appears to have produced the negative correlation between the two elements (Table 3). No other negative correlations were observed between Fe and other elements. On the other hand, the strongest positive correlations were found between Fe, Zn, and P. Zn and P concentrations in particular. Unlike Fe however, greater differences in Zn and P were observed between the locations than between the genotypes (Table 4).

**Table 3:** Pearson's correlation coefficient between the different trace elements in PBA HatTrick.

	**Ca**	**Mg**	**Na**	**K**	**P**	**S**	**Fe**	**Mn**	**Zn**	**B**	**Cu**
Ca	1										
Mg	0.618^*^	1									
Na	−0.352	−0.026	1								
K	0.544^*^	0.844^*^	0.120	1							
P	0.388	0.831^*^	0.453	0.735^*^	1						
S	0.061	0.644^*^	0.523^*^	0.469	0.905^*^	1					
Fe	0.439	0.498^*^	0.385	0.542^*^	0.673^*^	0.554^*^	1				
Mn	−0.542^*^	−0.839^*^	0.171	−0.745^*^	−0.691^*^	−0.498^*^	−0.474	1			
Zn	0.638^*^	0.439	0.192	0.329	0.626^*^	0.471	0.692^*^	−0.529^*^	1		
B	0.654^*^	0.945^*^	−0.295	0.816	0.669^*^	0.467	0.361	−0.886^*^	0.342	1	
Cu	0.095	−0.255	0.602^*^	−0.178	0.155	0.139	0.362	0.312	0.574^*^	−0.443	1

For Billa Billa, Roma, Warra, and Kingaroy, samples were n = 3 while NSW was n = 5. Grain samples from four locations within Queensland (Billa Billa, Warra, Roma, and Kingaroy) and a seed supplier from New South Wales were analyzed by ICP-OES. Values marked with an

* *Indicate a significant correlation between two elements (p < 0.05)*.

**Table 4:** Concentration of macro-elements in dry chickpea seed.

	**Ca (ppm)**		**Mg (ppm)**		**Na (ppm)**		**K (ppm)**		**P (ppm)**		**S (ppm)**	
**GENESIS090**												
Billa Billa	^efg^1420	(± 83.3)	^cdefg^1340	(± 17.3)	^cde^167.2	(± 31.0)	^bcde^10070	(± 115.5)	^abc^4000	(± 100.0)	^abcd^1930	(± 17.3)
Roma	^cdef^1600	(± 205.0)	^fg^1240	(± 10.0)	^cde^170.8	(± 27.3)	^bcde^10100	(± 458.3)	^efg^3330	(± 115.5)	^abcd^1870	(± 15.3)
Warra	^cdef^1640	(± 235.2)	^defg^1320	(± 52.9)	^cde^159.1	(± 38.8)	^fgh^9130	(± 305.5)	^ab^4100	(± 264.6)	^a^2040	(± 140.0)
**KALKEE**												
Billa Billa	^g^1050	(± 49.3)	^efg^1310	(± 10.0)	^cde^155.3	(± 18.4)	^ab^10800	(± 100.0)	^abc^3930	(± 57.7)	^abcd^1910	(± 36.1)
Roma	^fg^1240	(± 111.5)	^efg^1280	(± 0.00)	^cde^121.4	(± 18.9)	^a^11170	(± 57.7)	^def^3430	(± 115.5)	^bcd^1860	(± 45.1)
Warra	^fg^1210	(± 102.1)	^fg^1220	(± 124.9)	^cde^148.9	(± 105.6)	^defg^9400	(± 608.3)	^cde^3600	(± 264.6)	^abcd^1900	(± 87.2)
**MONARCH**												
Billa Billa	^fg^1270	(± 115.9)	^bcdef^1380	(± 20.8)	^a^633.3	(± 145.7)	^ab^10770	(± 251.7)	^bcde^3730	(± 57.7)	^cdef^1800	(± 15.3)
Roma	^cdef^1600	(± 200.3)	^fg^1220	(± 70.0)	^b^330.0	(± 65.6)	^bcde^10200	(± 100.0)	^g^2900	(± 200.0)	^defg^1760	(± 66.6)
Warra	^def^1570	(± 117.2)	^fg^1240	(± 34.6)	^bc^246.7	(± 30.6)	^gh^8630	(± 378.6)	^efg^3300	(± 264.6)	^ab^1980	(± 105.8)
**BOUNDARY**												
Billa Billa	^defg^1480	(± 262.1)	^bcde^1440	(± 105.0)	^cde^124.1	(± 36.3)	^abc^10470	(± 251.7)	^abcd^3870	(± 115.5)	^abcd^1890	(± 43.6)
Roma	^ab^2170	(± 57.7)	^bcde^1430	(± 55.1)	^cde^143.4	(± 15.5)	^ab^10970	(± 251.7)	^abcd^3830	(± 152.8)	^abcd^1910	(± 75.7)
Warra	^bcde^1920	(± 91.7)	^b^1520	(± 65.6)	^bcd^204.8	(± 42.0)	^fgh^9030	(± 230.9)	^abc^4030	(± 57.7)	^abc^1960	(± 40.4)
Kingaroy	^cdef^1650	(± 10.8)	^fg^1240	(± 21.0)	^de^83.1	(± 3.20)	^gh^8570	(± 60.8)	^k^1860	(± 35.4)	^fg^1610	(± 17.9)
**CICA0912**												
Billa Billa	^def^1590	(± 62.4)	^b^1520	(± 50.0)	^de^73.2	(± 12.4)	^abc^10570	(± 450.9)	^abc^3970	(± 115.5)	^cdef^1810	(± 25.2)
Warra	^abc^2100	(± 355.0)	^bcd^1490	(± 101.5)	^cde^108.2	(± 17.3)	^fgh^8900	(± 400.0)	^ab^4130	(± 152.8)	^abc^1960	(± 51.3)
Kingaroy	^bcde^1850	(± 68.0)	^g^1180	(± 41.8)	^e^40.0	(± 1.10)	^gh^8710	(± 196.8)	^k^2290	(± 176.5)	^def^1780	(± 69.4)
**HATTRICK**												
Billa Billa	^bcd^1960	(± 163.7)	^bc^1510	(± 20.0)	^cde^160.2	(± 62.2)	^cdef^9770	(± 550.8)	^cde^3630	(± 251.7)	^cdef^1800	(± 20.8)
Roma	^a^2570	(± 152.8)	^bcde^1440	(± 5.80)	^de^71.0	(± 14.5)	^efg^9300	(± 100.0)	^fg^3030	(± 57.7)	^efg^1680	(± 30.6)
Warra	^bcd^1960	(± 315.3)	^b^1530	(± 85.0)	^cde^165.6	(± 14.9)	^fgh^9030	(± 57.7)	^a^4200	(± 100.0)	^abcd^1900	(± 10.0)
Kingaroy	^bcdef^1670	(± 129.6)	^fg^1240	(± 47.8)	^e^51.5	(± 0.40)	^h^8360	(± 60.0)	^k^1990	(± 44.0)	^fg^1650	(± 30.5)
NSW	^a^2420	(± 109.5)	^a^1800	(± 46.2)	^e^63.4	(± 6.30)	^bcd^10160	(± 230.2)	^ab^4080	(± 83.7)	^cde^1820	(± 27.9)

*Data are presented as a mean ± SD. All cultivars from had n = 3 except for HatTrick from NSW, which has n = 5. Values sharing the same superscript letters indicate groups that are not significantly different at p < 0.05 when tested with one-way ANOVA, using Tukey's HSD post-hoc test*.

### Cotyledons serve as the primary store for Fe in PBA hattrick seeds

**Figure 2:**
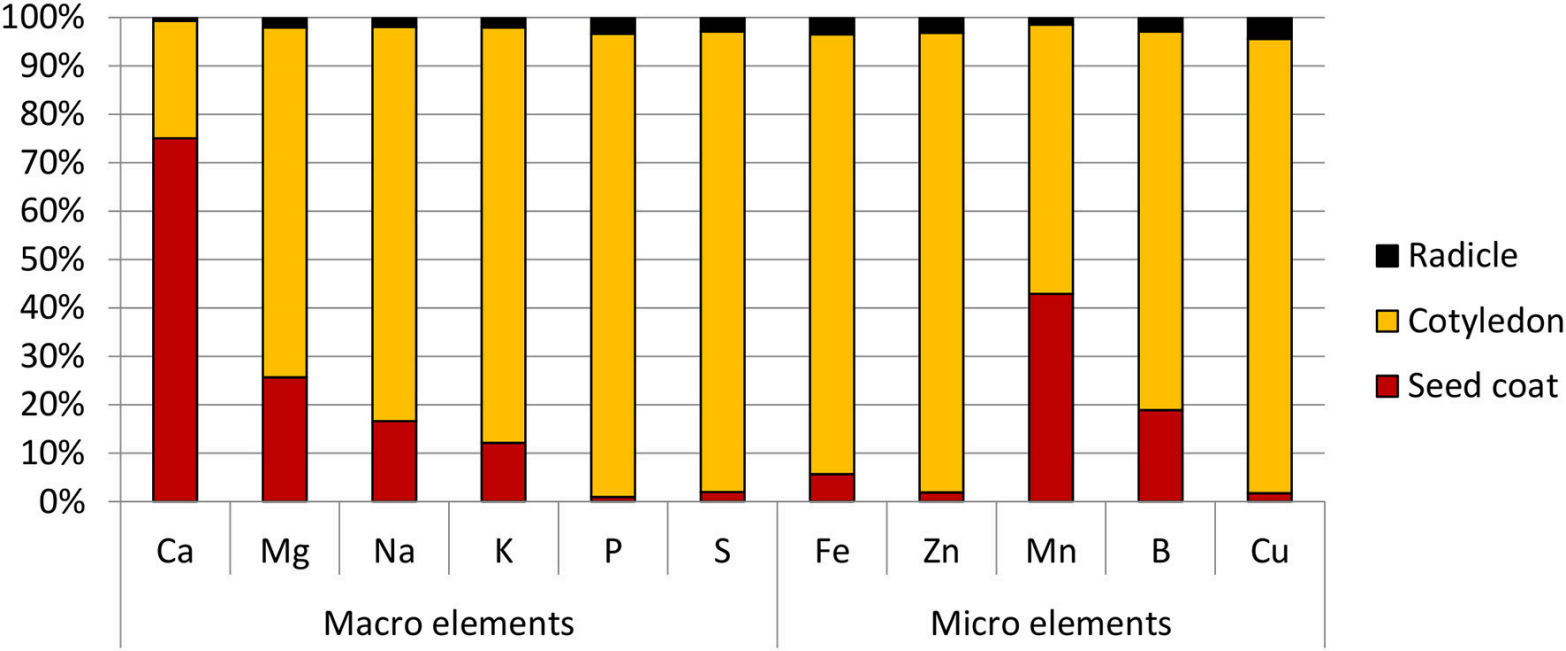
Distribution of macro and micro elements in chickpea (cv HatTrick) seed expressed as a percentage of the total element content in the three main tissue types present in the grain. Values for the elemental profile was derived from a bulked flour sample produced from pooling tissue from 100 seeds. Three technical replicates were used. Mass distribution in the seed was calculated from 10 biological replicates.

Amongst the different part of the seed, the radicle was found to have the highest Fe concentration at 95.0 ppm, followed by the cotyledons at 50.0 ppm. Due to its small mass however, its contribution only 3% to the total seed Fe content. The cotyledons on the other hand, constituted the bulk of the seed mass and contained 90% of the seed's total Fe. It was also the main store for all the other elements tested. The exception to this was calcium and manganese—the bulk of the former was found in the seed coat, while the latter was almost equally divided between the seed coat and cotyledons (Figure 2).

### Legume NAS homologs form distinct branches among the non-graminaceous orthologues

Concerning their chromosomal locations, the CaNAS2 and CaNAS4 genes were located on chromosomes 4 (Ca4) and 3 (Ca3) respectively, while both CaNAS1 and 3 were located on chromosome 1 (Ca1). All four CaNAS genes were found to be to consist of a single exon. CaNAS1, 2, 3, and 4 coded for 285, 306, 311, and 318 amino acids respectively. In the CaNAS1 protein, a longer N-terminal and a shorter C-terminal was seen compared to the other three CaNAS homologs.

**Figure 3:**
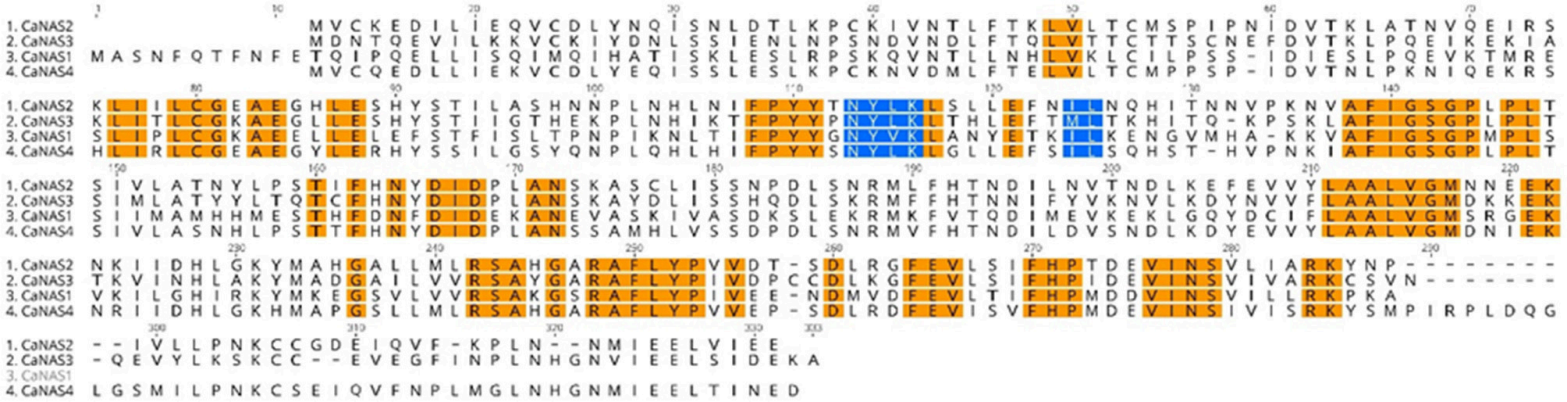
Amino acid sequence alignment of the four chickpea CaNAS amino acid sequences. Orange sections indicate conserved regions. Blue sections represent the YXXΦ and the LL, IL, or ML motifs.

In the four CaNAS amino acid sequences, several highly conserved regions were noted (Figure 3). Of these, the YXXΦ (Y refers to tyrosine, X to any amino acid residue, and Φ to bulky hydrophobic residues) and di-leucine (LL; leucine may be substituted with isoleucine) motifs were known to be conserved amongst the NAS homologs. Amongst some of the legume NAS sequences however, a variation of the LL motif was observed where the first leucine was substituted by methionine. This was seen in the CaNAS3 protein sequence, as well as in MtNAS2 from *Medicago truncatula* and LjNAS2 from *Lotus japonicus* (Supplementary Table 3).

**Figure 4:**
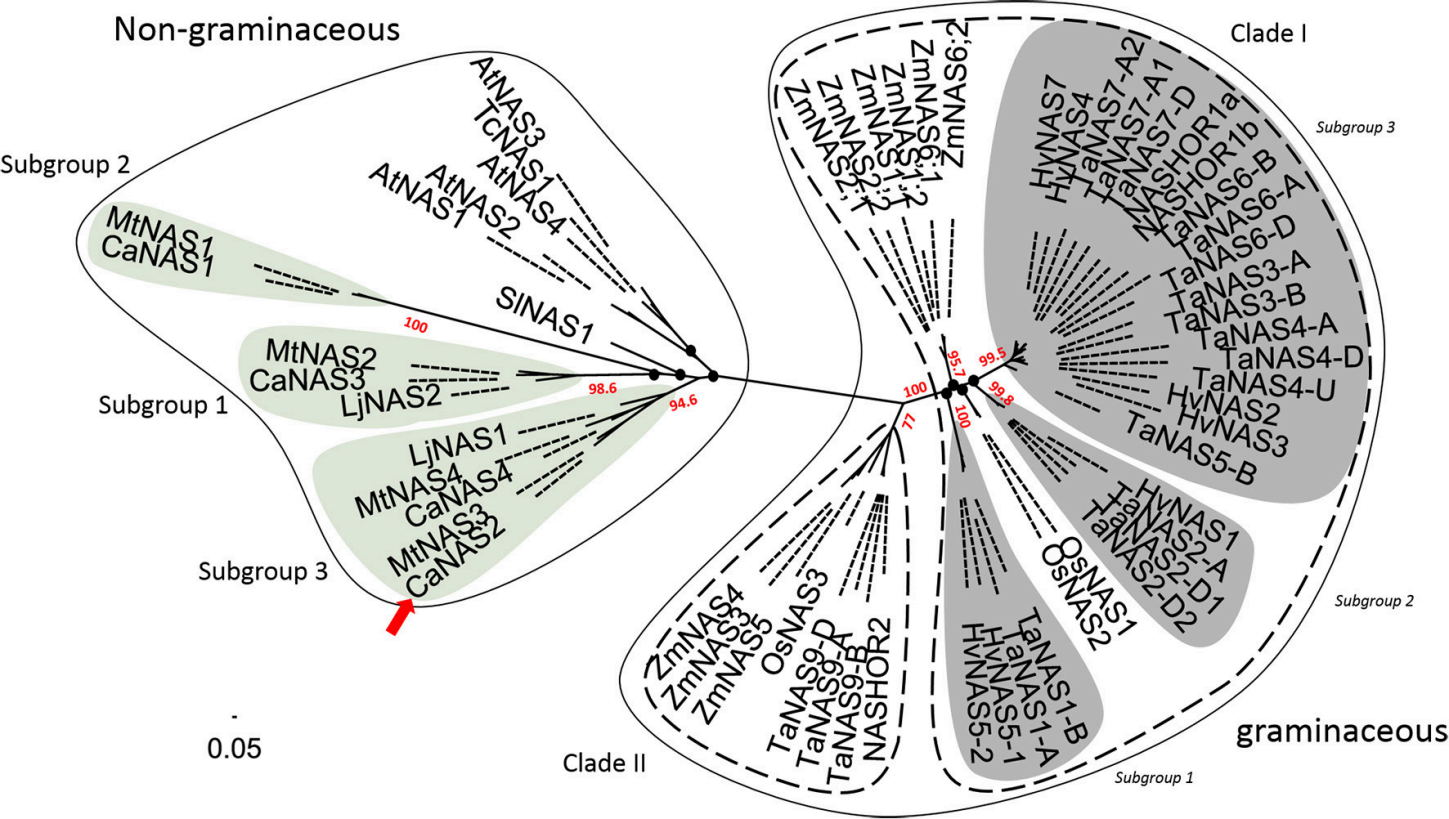
Phylogenetic relationship between NAS proteins from plants. The scale bar and branch labels represent the number of substitutions per site. The legume-specific group are as indicated by the green circles and the red arrow shows the position of CaNAS2 in the unrooted phylogenetic tree. Species included in this tree are *Arabidopsis thaliana* (AtNAS), *Hordeum vulgare* (HvNAS), *Lotus japonicus* (LjNAS), *Medicago truncatula* (MtNAS), *Oryza sativa* (OsNAS), *Thlaspi caerulescens* (TcNAS), *Solanum lycopersicum* (SlNAS), *Zea mays* (ZmNAS), and *Triticum aestivum* (TaNAS). Black nodes (•) represent weak bootstrap values (< 75%). The scale bar corresponds to branch length and longer branches correspond to greater numbers of nucleic acid polymorphisms along the sequence.

Phylogenetic analysis of the 58 NAS proteins revealed a clear distinction between graminaceous and non-graminaceous sequences (Figure 4). Two clades were present in the former and were consistent with a prior report (Bonneau et al., 2016). With the latter, three subgroups for legumes were observed—these were defined as subgroups 1, 2, and 3. Subgroup 1 consisted of includes CaNAS1 from chickpea and MtNAS1 from *Medicago truncatula*. Subgroup 2 consisted of CaNAS3, MtNAS2, and LjNAS2 (from *Lotus japonicus*). Lastly, Subgroup 3 consisted of CaNAS2 and 4, MtNAS3 and 4, and LjNAS1. The presence of CaNAS2/MtNAS3 and CaNAS4/MtNAS4 in the same branch is most likely due to genome duplication.

Further bioinformatic analysis of the CaNAS2 protein indicated an approximate molecular weight and pI of 34.36kDA 5.52 respectively. The enzyme was mostly hydrophilic with a potential transmembrane domain at position 126–151. A non-cytoplasmic localisation was predicted, though no apparent signaling peptides were detected.

### CaNAS2 is expressed in various vegetative tissues under Fe sufficient conditions

**Figure 5:**
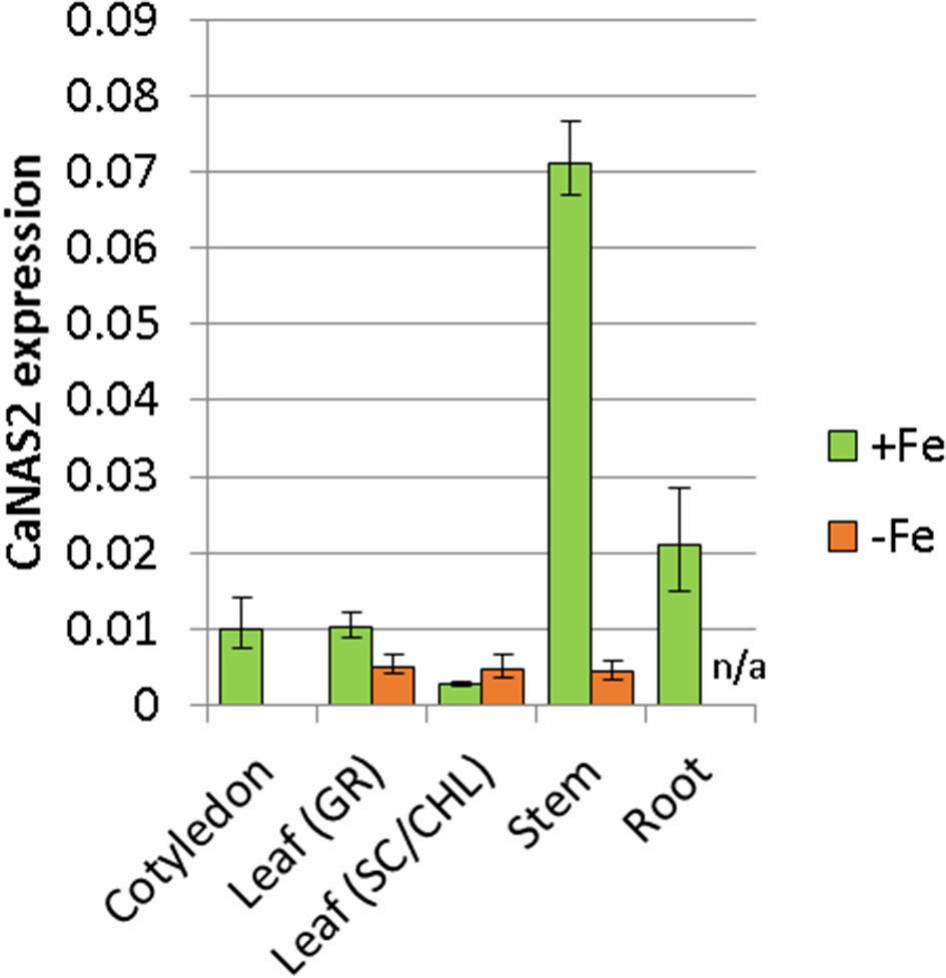
Expression of CaNAS2 in different tissues under Fe-sufficient (+Fe) and Fe-deficient conditions (–Fe) measured via quantitative real-time PCR. Each data point is an average of three biological replicates; each of which was derived from three technical replicates. Error bars represent standard error. Expression was normalisedto the housekeeping genes *CaGAPDH* and *CaEF1*α. No viable RNA could be extracted from the Fe-deficient root samples; they were therefore excluded from this analysis. Leaf (GR), Green leaf, Leaf (SC/CHL), Senescing leaf (for Fe sufficient plants), and chlorotic leaf (for Fe deficient plants).

The expression pattern of CaNAS2 was examined under Fe-sufficient and Fe-deficient conditions. The Fe-deficient plants used in this study were observed to be paler green than the Fe sufficient controls, with severe chlorosis in the young leaves. No nodules were observed in either Fe-deficient or Fe-sufficient plants. CaNAS2 transcripts were detected in all the tissue types tested, though the levels were largely influenced by Fe status, with an overall downregulation under Fe deficiency. Gene expression in the Fe deficient plants, where detected, was generally low, and comparable across all tissues (Figure 5). Similar levels were also detected in the senescing leaf of Fe-sufficient plants. By contrast, other Fe sufficient tissues exhibited markedly higher expression, particularly in the stem, cotyledons, and roots. A 16-fold difference was seen between stems of the Fe-sufficient and Fe deficient plants, while expression was only detected in Fe-sufficient cotyledons. For root tissue however, no data could be obtained for the Fe deficient plants due to the consistently poor quality of the extracted RNA.

### Transgenic chickpea highly express both transgenes and have increased Fe in leaf tissue and increased nicotianamine in seed tissue

**Figure 6:**
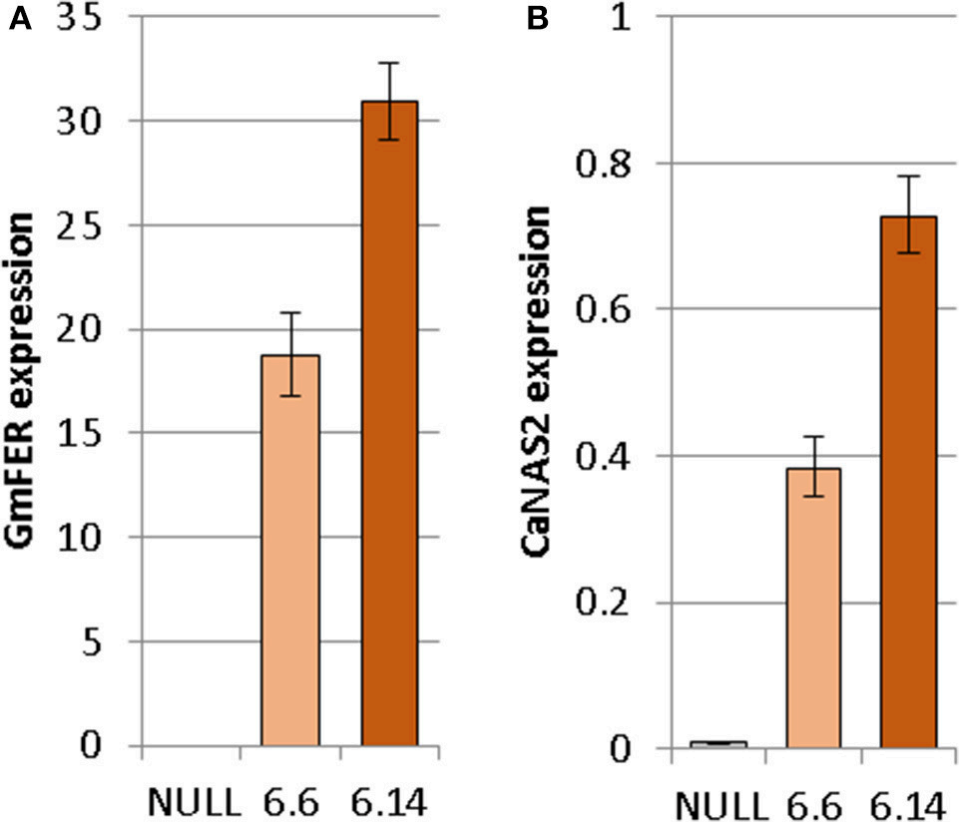
Expression of **(A)** GmFER and **(B)** CaNAS2 in the null and GE chickpea events normalized to the housekeeping genes *CaGAPDH* and *CaEF1*α through quantitative real-time PCR. Each data point is an average of three biological replicates; each of which was derived from three technical replicates. Error bars indicate standard error.

**Figure 7:**
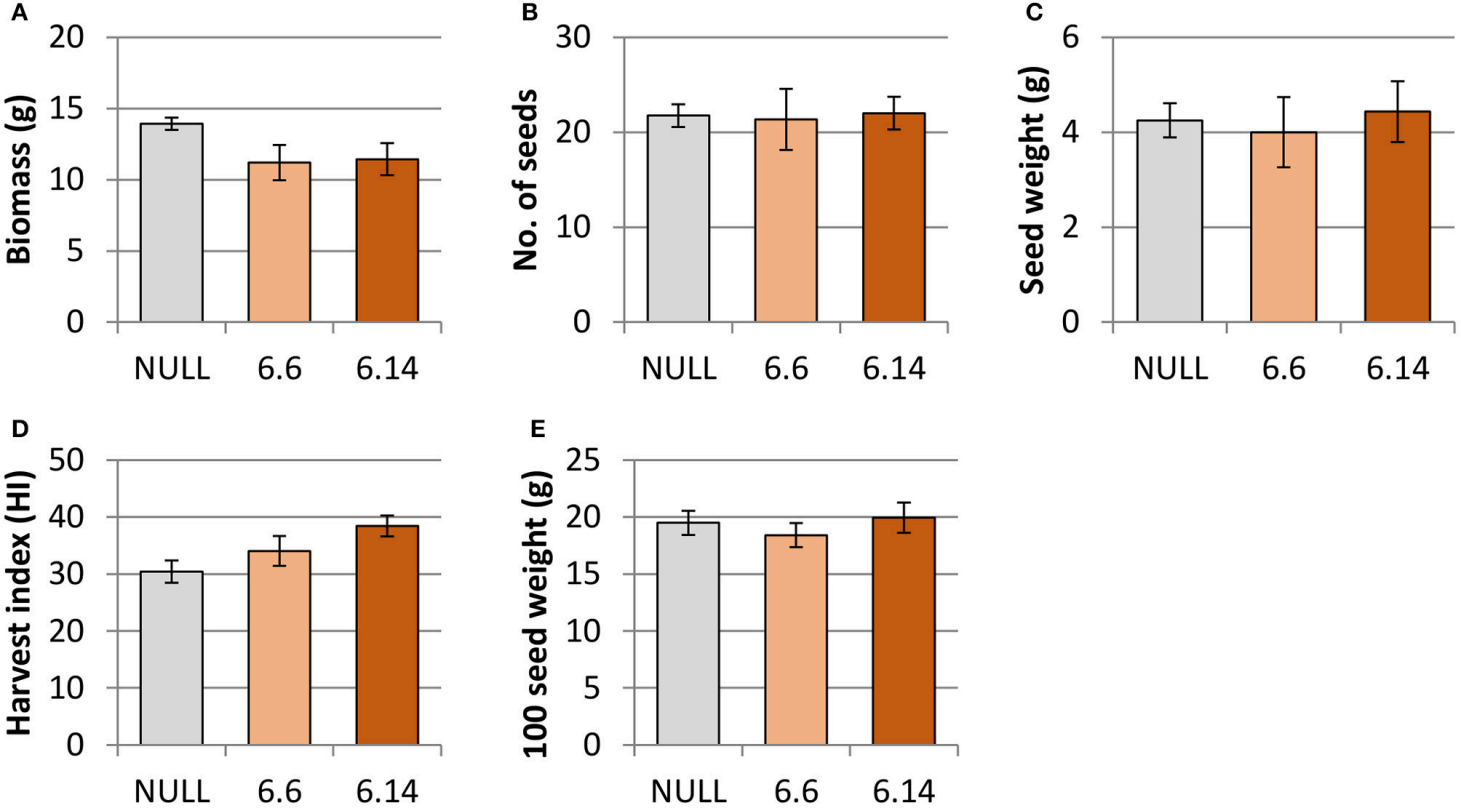
Agronomic characteristics of the null and GE chickpea under glasshouse conditions. **(A)** biomass; **(B)** number of seeds; **(C)** seed weight; **(D)** harvest index; **(E)** 100 seed weight. Null (n = 4), Event 6.6 (n = 9) and Event 6.14 (n = 14). Each data point represent a mean of all biological replicates, and error bars indicate standard error. No significant differences (p ≤ 0.05) were observed between the null and GE lines when tested with one-way ANOVA, using Dunnett's post-hoc test.

**Figure 8:**
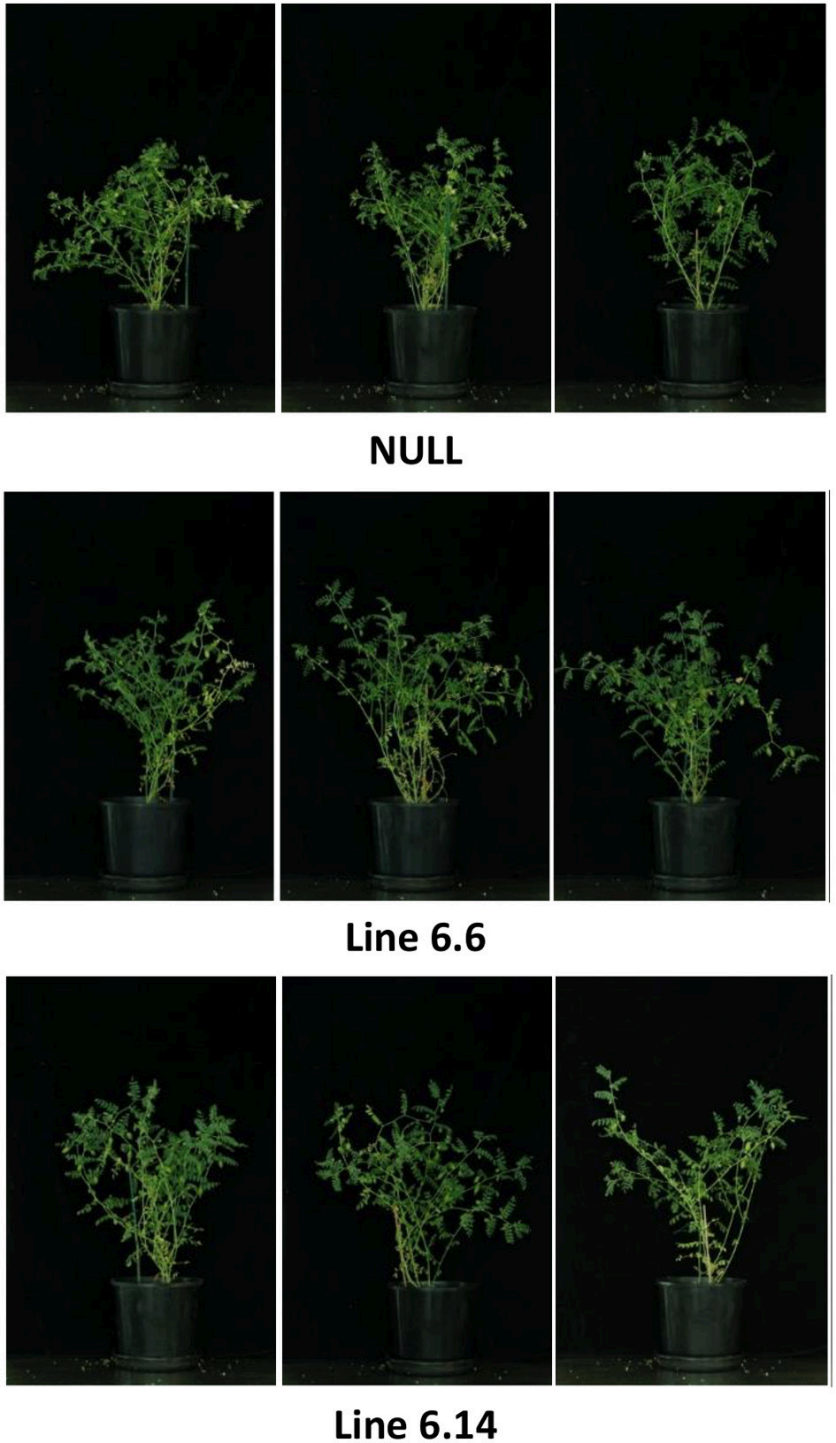
Morphologically similar 9 week old null and GE chickpea at the flowering/pod-filling stage.

Both of the regenerated GE events (6.1 and 6.14) that were propagated to the T_3_ generation were confirmed via Southern blot to have single transgene integration sites. Expression analyses showed a 49- and 93-fold increase in *CaNAS2* expression in events 6.6 and 6.14, respectively, compared to null segregant controls (Figure 6B). Expression analysis showed an 18- and 30-fold increase in expression of *GmFER* in events 6.6 and 6.1, respectively, compared to null segregant controls (Figure 6A). Agronomic performance of the T_3_ GE events in the glasshouse was generally comparable to the null controls and no significant differences were seen in terms of morphology and the other parameters measured (Figures 7, 8).

**Figure 9:**
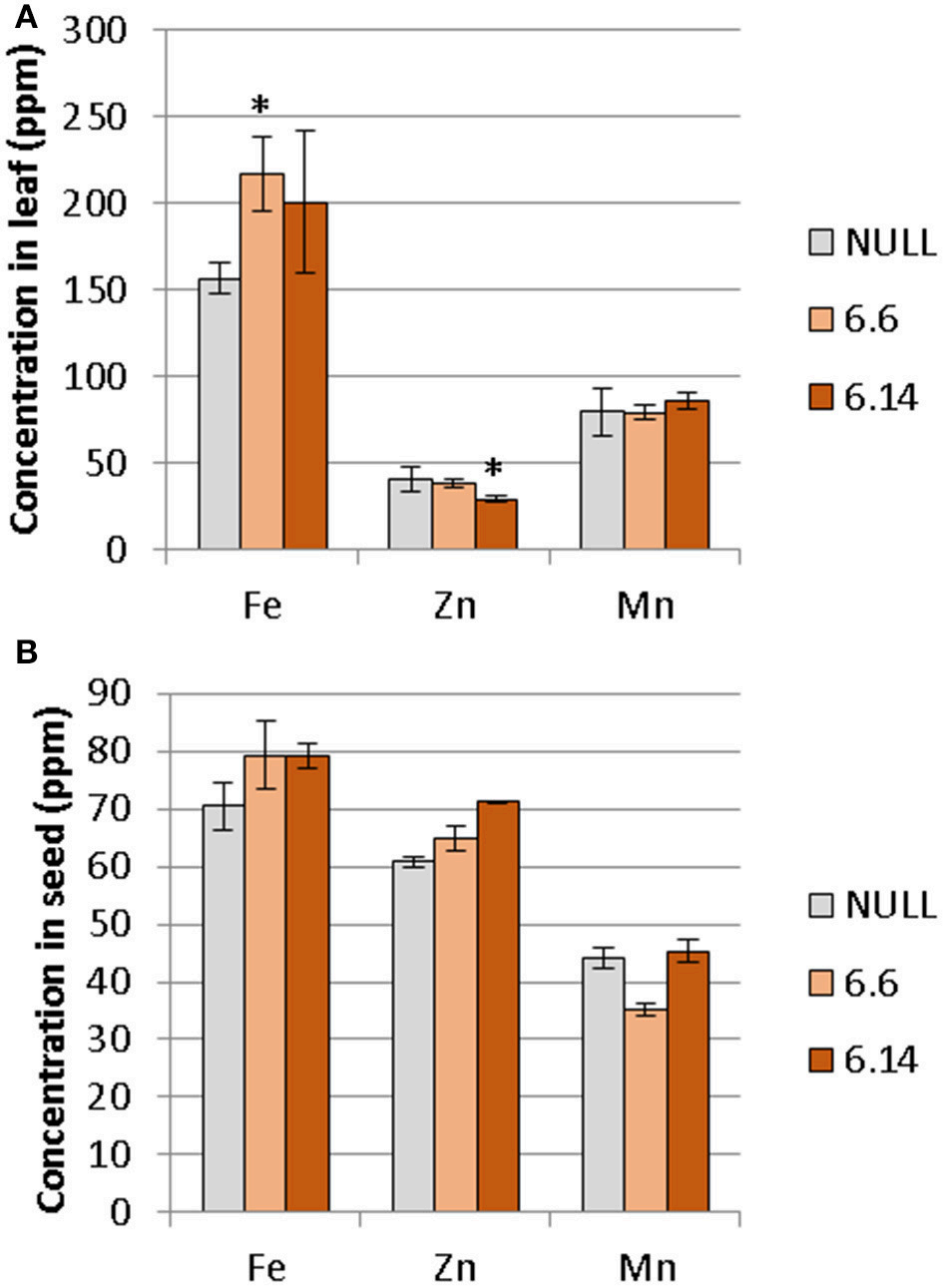
Mean concentration of iron, zinc, and manganese in the **(A)** leaves and **(B)** seeds of the null and GE chickpea lines. For the leaves, the null had n = 5, ^*^Event 6.6 had n = 14, and Event 6.14 had n = 3. For the seeds, the null and Event 6.6 had n = 4, while Event 6.14 had n = 4. ^*^Indicates significant difference compared to the null when tested with one-way ANOVA, using Dunnett's post-hoc test (p ≽ 0.05).

**Figure 10:**
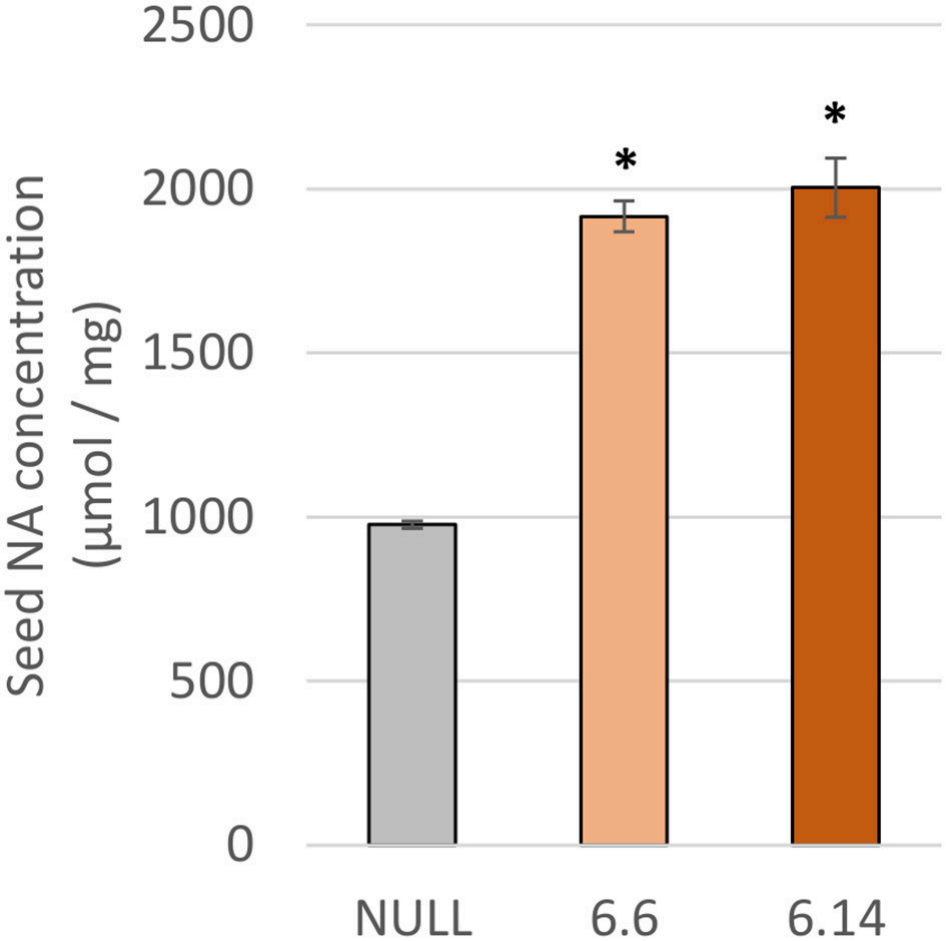
NA concentration in the null and GE chickpea events. For each transgenic event, four technical replicates were drawn from a bulked flour sample produced from the pooled seeds of three plants. ^*^Indicates significant difference compared to the null when tested with one-way ANOVA, using Dunnett's post-hoc test (p ≤ 0.001).

Two differences were observed with respect to micronutrient composition of the leaf tissue of events 6.6 and 6.14 compared to null segregant controls (Figure 9). Event 6.6 had significantly higher leaf Fe concentration which was 1.39-fold higher than the null segregant control. Event 6.14 had significantly lower leaf Zn concentration which was 1.4-fold lower than the null segregant control. No significant differences for Fe, Zn, or Mn concentrations were observed in the seed of events 6.6 and 6.14. Seed from events 6.6 and 6.14 contained significantly higher NA concentration which was nearly 2-fold higher than the null segregant control (Figure 10).

## Discussion

### Conditions affecting seed Fe concentrations in chickpea and the selection of cv PBA hattrick for Fe biofortification research

Australian-grown chickpeas were previously reported to contain up to 140 ppm of Fe in their seed, though average values were ~50 ppm (Petterson and Mackintosh, 1994). Such average values appear to be the norm globally, with similar results reported in chickpea from other countries (Meiners et al., 1976; Jambunathan and Singh, 1981; Thavarajah and Thavarajah, 2012; Diapari et al., 2014; Ray et al., 2014). That similar average values for seed Fe concentrations were obtained for the six cultivars used in our study, indicating that the cultivars used fell within that global norm.

A past study by Ray et al. (2014) has reported seed Fe concentration to be influenced firstly by environmental conditions, then by genotype. Our observations were only partially consistent with that report, perhaps due to the smaller number of locations and cultivars we examined. Between the cultivars and most locations, no major differences were noted. The major environmental effect on seed Fe was only observed where soil Fe bioavailability was potentially compromised, such as with the Kingaroy samples which had lower seed Fe concentrations. Past records have shown Kingaroy ferrosols to be acidic with a high manganese to Fe ratio, and such conditions have been documented to inhibit Fe uptake, sometimes to the point of chlorosis (Twyman, 1951; Tanaka and Navasero, 1966; Alvarez-Tinaut et al., 1980). In our study, this inhibition was asymptomatic; no Fe deficiency symptoms were reported by the growers, and the effect was only apparent upon assessment of the seed mineral profile. This is potentially problematic where biofortification efforts are concerned as attempts to increase Fe concentrations may be unknowingly hijacked by adverse soil conditions.

An environmental effect was also apparent in the Zn and P concentrations, both of which were positively correlated with Fe and affect seed nutritional value. With Zn, significant variations were noted between all sites regardless of cultivar, corresponding with observations by other authors who also reported significant year to year variations, even with seed from the same sites (Diapari et al., 2014; Ray et al., 2014). Management practices may explain at least part of the higher degree of Zn variation compared to Fe. Application of Zn fertilizers is a recommended practice in chickpea cultivation due to the risk of Zn deficiency in most Australian soils (Norton, 2013; Pulse Australia, 2016). The effects of Zn fertilization on grain Zn concentration however, are unpredictable and may differ between seasons (Akay, 2011). This variability is compounded by the effect of other management regimes like the application of P fertilizer. Aside from directly affecting grain P concentration (Saastamoinen, 1987), studies in pearl millet and wheat have highlighted a negative impact of P fertilizer on seed Zn concentration (Buerkert et al., 1998; Ryan et al., 2008). The precise reason behind it is uncertain, though it has been attributed to altered zinc uptake and the dilution effect caused by increased yields. The former was deemed the more likely, given the absence of adverse effects on seed Fe concentration (Ryan et al., 2008), though regardless, the implications on the nutritional quality of the seeds are still considerable. Seed P is primarily stored as phytate (Lolas et al., 1976; Griffiths and Thomas, 1981; Ravindran et al., 1994), a potent inhibitor of Fe and zinc bioavailability (Turnlund et al., 1984; Sandberg et al., 1989, 1999), and its levels can be considered a crude indicator of micronutrient bioavailability.

In terms of the genetic effect, only a slight influence was seen. Few significant differences were found amongst the cultivars assessed, though kabuli cultivars had marginally higher Fe concentrations than the desi. This lack of difference is likely a product of the breeding process. Currently, no information exists for micronutrient accumulating traits in existing germplasm. As micronutrient accumulation is often accompanied by yield penalties (Garvin et al., 2006; Ficco et al., 2009; Diapari et al., 2014), it is likely that such traits may have been bred out of the current cultivars as breeding efforts in Australia have primarily focused on yield, abiotic stress, and biotic stress resistance with no consideration for nutritional value (Pulse Breeding Australia, 2017). Consequently, reintroduction of Fe-accumulation traits may prove challenging, though the difficulty can be alleviated with modern biotechnology.

For this purpose the cultivar with the lowest Fe concentrations, PBA HatTrick, was identified as a suitable candidate for Fe biofortification. The benefits of this choice are manifold. PBA HatTrick is a popular choice amongst growers due to its high yield and resistance to phytophthora root rot. As a desi cultivar, it also has great potential for widespread dissemination, as desi constitutes 90% of the Australian chickpea export and therefore the bulk of the international market (Pulse Australia, 2016). With Fe localized primarily to the cotyledon, which is the main product, enhancements in Fe concentration will reach the consumer regardless of the form in which the seed is consumed. Bioavailability, however, may be a concern as phosphorus (and by extension, phytate) co-localizes with Fe to the cotyledons. This may perhaps be addressed with appropriate biofortification strategies and choice of target genes, one of which, for the purposes of this study, has identified as the novel CaNAS2 gene.

### CaNAS2 has a potential housekeeping role under Fe sufficient conditions

In our study, CaNAS2 grouped with the other dicot sequences in a separate clade to monocot sequences. This dichotomy between the dicot and monocot sequences is consistent with the findings of other authors (Hakoyama et al., 2009; Filipe de Carvalho et al., 2012), and is likely reflective of the differing physiological roles of NAS between the two. However whether this is conclusive remains to be seen due to the limited number of experimentally verified homologs; monocot sequences used in this study were of graminaceous origins, and the inclusion of the non-graminaceous homologs may potentially alter the existing arrangment. Nonetheless recent evidence have revealed functional grouping amongst NAS homologs (Bonneau et al., 2016) in terms of roles in development and Fe deficiency. Assuming this is universal amongst higher plants, such grouping will allow for accurate prediction of the function of closely related homologs. This accuracy however, remains subject to the availability of sequences that can be extrapolated from–such is evident in this study. For example, nodule-specific expression of LjNAS2 (Hakoyama et al., 2009) was neither mirrored in the closely related MtNAS2 (Medicago truncatula Gene Expression Atlas, 2014), nor in the any of the other legume NAS used in this study. Whether the third member of Subgroup 2, CaNAS3, will be nodule-specific is uncertain—this could not be investigated in our study due to the absence of nodules as the plants were not inoculated with *Rhizobium*. Still, given that the substitution of the di-leucine motif by a methionine is a trait unique to Subgroup 2, it is plausible that some functional specialization is present. Further investigation and discovery of more nodule-specific homologs may shed more light on this.

Functional specialization may also be present in Subgroup 1, to which CaNAS1 and MtNAS1 belong. The nature of this specialization is still inconclusive. Subgroup 1 was the most divergent from other NAS proteins used in the phylogenetic analysis and no discernible trend could be seen between members. No orthologue of CaNAS1 was found in *Lotus japonicus*, perhaps due to the genome evolution in legumes (Wang et al., 2017). As with Subgroup 2, further study is required before conclusive statements may be made.

In the interim, only Subgroup 3 bears enough information for reasonable inference of function. Subgroup 3 homologs are notable for their widespread expression in various vegetative tissues (Hakoyama et al., 2009; Medicago truncatula Gene Expression Atlas, 2014), and such is also seen in CaNAS2 and 4 (Figure 5, Supplementary Figure 1). While the expression sites vary between homologs, ranging from roots, to leaves, and cotyledons, a common feature is the expression in the stem. Using LjNAS1 as a reference, this pattern may indicate a housekeeping role in the systemic redistribution of Fe (Hakoyama et al., 2009) which, in the case of CaNAS2, occurs under Fe-sufficient conditions. It is also likely that the expression of NAS in the diverse sites may operate at different scales, given the involvement of NA in long and short-distance translocation. Expression in the stem may serve to feed NA into the vascular tissue and symplast for systemic transport, while expression in the other locations may provide NA for more localized translocation.

Concerning the movement and localization of CaNAS2 within an intracellular context, the results predicted a non-cytoplasmic, and potentially vesicular, localization. The accuracy of this however, is contentious. The YXXΦ and LL motifs conserved in the NAS family have been linked to maintenance of enzyme structure, and vesicular localization and movement (Nozoye et al., 2014a). Studies of various NAS homologs have yielded conflicting results. For example, vesicular localization have only been confirmed in OsNAS2, ZmNAS1 and ZmNAS2, while ZmNAS3 and the AtNAS family, localized to the cytoplasm (Mizuno et al., 2003; Nozoye et al., 2014a,b). It was proposed by Nozoye et al. (2014b) that vesicular localization is required for DMA synthesis, hence its specificity to the graminaceous homologs. With chickpea lacking in that regard, its localization pattern is likely to be more similar to that of AtNAS, though more studies are required to confirm this.

### Constitutive expression of CaNAS2 and GmFER does not increase seed Fe concentration but is likely to increase seed Fe bioavailability

As demonstrated in this study, constitutive overexpression of the endogenous *CaNAS2* gene combined with constitutive expression of the *GmFER* gene in chickpea resulted in higher leaf Fe in one event, and lower leaf Zn in the other event, with no apparent effects on yield or morphology in either event. Seed Fe, Zn and Mn concentrations were not changed in either event. These results suggest that high expression of the *CaNAS2* and *GmFer* transgenes is not an effective strategy for improving the micronutrient composition of chickpea grain. Potentially a better strategy in the future would be to use a seed-specific promoter to drive *GmFer* expression in conjunction with *CaNAS2* overexpression. Indeed, this method has been demonstrated to be extremely effective in rice, producing a 7.5-fold increase in the Fe concentration of polished seeds with no yield penalty (Trijatmiko et al., 2016).

Due to high levels of inhibitory compounds (i.e., phytic acid), the Fe bioavailability in pulses is low relative to other crops (Hemalatha et al., 2007). As NA is a known promoter of Fe bioavailability, doubling the concentration of NA in chickpea flour may increase Fe bioavailability without alterations to seed mineral concentration (Zheng et al., 2010; Eagling et al., 2014). Future *in vitro* Fe bioavailability studies utilizing the Caco-2 cell line assay are needed to confirm increased Fe bioavailability in high-NA chickpea events.

## Author contributions

SM, BW, SD, and AJ conceived and designed the project. GT, SD, HL, AC, TH, MK, AJ, JPB, and JTB designed and performed the experiments. GT, JTB, and JPB data analysis. GT, JTB, JPB, and AJ wrote the paper.

### Conflict of interest statement

AJ is an editor for the Improving the Nutritional Content and Quality of Crops: Promises, Achievements, and Future Challenges topic of Frontiers in Plant Science. The remaining authors declare that the research was conducted in the absence of any commercial or financial relationships that could be construed as a potential conflict of interest.

## Footnotes

^1^Available online at http://www.genome.jp/).
