# Supplementary material for: Investigation of Baseline Iron Levels in Australian Chickpea and Evaluation of a Transgenic Biofortification Approach
Source: Front Plant Sci. 2018 Jun 14;9:788. doi: 10.3389/fpls.2018.00788 (PMC6010650; doi:10.3389/fpls.2018.00788)
Supplement: Supplementary file 1 [file Table_1.DOCX]

Supplementary Material

Investigation of baseline iron levels in Australian chickpea and evaluation of a transgenic biofortification approach

Tan, Z.H.G.^1^, Das Bhowmik, S.S.^1^, Hoang, T.M.L.^1^, Karbaschi, M.R.^1^, Long, H.^1^, Cheng, A.^1^, Bonneau, J.P. ^2^, Beasley, J.T.^2^, Johnson, A.A.T.^2^, Williams, B.^1^, Mundree, S.G.^1^*

^1^Centre for Tropical Crops and Biocommodities, Queensland University of Technology, Queensland, Australia

^2^School of Biosciences, University of Melbourne, Victoria, Australia

*** Correspondence:** Prof Sagadevan Mundree: sagadevan.mundree@qut.edu.au

Supplementary Table 1. Cultivation conditions for each chickpea growing location.

| **Site** | **Australian soil classification** | **Total fertiliser used** | **Approximate rainfall during growing period (mm)** |
| --- | --- | --- | --- |
| Billa Billa | Gray vertosol | 25kg/ha Zinz star 25 | 64.5 |
| Roma | Black vertosol | 25kg/ha Zinz star 25 | 85 |
| Warra | Gray vertosol | 25kg/ha Zinz star 25 | 80.2 |
| Kingaroy | Ferrosol | n/a | 594 |
| NSW | n/a | n/a | n/a |
