# Supplementary material for: Investigation of Baseline Iron Levels in Australian Chickpea and Evaluation of a Transgenic Biofortification Approach
Source: Front Plant Sci. 2018 Jun 14;9:788. doi: 10.3389/fpls.2018.00788 (PMC6010650; doi:10.3389/fpls.2018.00788)
Supplement: Supplementary file 2 [file Table_2.DOCX]

Supplementary Material

Investigation of baseline iron levels in Australian chickpea and evaluation of a transgenic biofortification approach

Tan, Z.H.G.^1^, Das Bhowmik, S.S.^1^, Hoang, T.M.L.^1^, Karbaschi, M.R.^1^, Long, H.^1^, Cheng, A.^1^, Bonneau, J.P. ^2^, Beasley, J.T.^2^, Johnson, A.A.T.^2^, Williams, B.^1^, Mundree, S.G.^1^*

^1^Centre for Tropical Crops and Biocommodities, Queensland University of Technology, Queensland, Australia

^2^School of Biosciences, University of Melbourne, Victoria, Australia

*** Correspondence:** Prof Sagadevan Mundree: sagadevan.mundree@qut.edu.au

Supplementary Table 2. Percentage of similarity between the CaNAS and OsNAS amino acid sequences.

|  | **OsNAS1** | **OsNAS2** | **OsNAS3** |
| --- | --- | --- | --- |
| **CaNAS2 (XP_004495658.1)** | 44.984 | **44.984** | 47.249 |
| **CaNAS (XP_004487761.1)** | 40.741 | 40.062 | 42.154 |
| **CaNAS (XP_004488704.1)** | 43.262 | 42.908 | 42.215 |
| **CaNAS (XP_004494544.1)** | 43.168 | 43.168 | 47.205 |
