# Supplementary material for: Investigation of Baseline Iron Levels in Australian Chickpea and Evaluation of a Transgenic Biofortification Approach
Source: Front Plant Sci. 2018 Jun 14;9:788. doi: 10.3389/fpls.2018.00788 (PMC6010650; doi:10.3389/fpls.2018.00788)
Supplement: Supplementary file 3 [file Table_3.DOCX]

Supplementary Material

Investigation of baseline iron levels in Australian chickpea and evaluation of a transgenic biofortification approach

Tan, Z.H.G.^1^, Das Bhowmik, S.S.^1^, Hoang, T.M.L.^1^, Karbaschi, M.R.^1^, Long, H.^1^, Cheng, A.^1^, Bonneau, J.P. ^2^, Beasley, J.T.^2^, Johnson, A.A.T.^2^, Williams, B.^1^, Mundree, S.G.^1^*

^1^Centre for Tropical Crops and Biocommodities, Queensland University of Technology, Queensland, Australia

^2^School of Biosciences, University of Melbourne, Victoria, Australia

*** Correspondence:** Prof Sagadevan Mundree: sagadevan.mundree@qut.edu.au

Supplementary Table 3. List of genes used in phylogenetic analyses. The species, the gene name, the Genbank identifier for coding DNA sequence and protein, and the Di-leucine motif.

| Species | Gene name | Genbank CDS ID | Genbank Protein ID | Di-leucine motif |
| --- | --- | --- | --- | --- |
| Cicer arietinum | *CaNAS2* | XM_004495601.2 | XP_004495658.1 | IL |
| Cicer arietinum | *CaNAS3* | XM_004487704.2 | XP_004487761.1 | ML |
| Cicer arietinum | *CaNAS1* | XM_004488647.2 | XP_004488704.1 | IL |
| Cicer arietinum | *CaNAS4* | XM_004494487.2 | XP_004494544.1 | IL |
| Medicago truncaluta | *MtNAS1* | XM_013608903.1 | XP_013464357.1 | IL |
| Medicago truncaluta | *MtNAS2* | XM_003594705.1 | XP_003594753.1 | ML |
| Medicago truncaluta | *MtNAS3* | XM_003591172.2 | XP_003591220.1 | IL |
| Medicago truncaluta | *MtNAS4* | XM_013595007.1 | XP_013450461.1 | IL |
| Lotus japonicus | *LjNAS1* | AB480829.1 | BAH22562.1 | IL |
| Lotus japonicus | *LjNAS2* | AB480830.1 | BAH22563.1 | ML |
| Arabidopsis thaliana | *AtNAS1* | NM_120577.4 | NP_196114.1 | LL |
| Arabidopsis thaliana | *AtNAS2* | NM_124990.2 | NP_200419.1 | LL |
| Arabidopsis thaliana | *AtNAS3* | NM_100794.4 | NP_172395.1 | LL |
| Arabidopsis thaliana | *AtNAS4* | NM_104521.3 | NP_176038.1 | LL |
| Thlaspi caerulescens | *TcNAS1* | AJ300446.1 | CAC82913.1 | LL |
| Solanum lycopersicum | *SlNAS1* | NM_001309378.1 | NP_001296307.1 | IL |
| Hordeum vulgare | *HvNAS1* | AB010086 | BAA74580.1 | LL |
| Hordeum vulgare | *HvNAS2* | AB011265 | BAA74582.1 | LL |
| Hordeum vulgare | *HvNAS3* | AB011264 | BAA74581.1 | LL |
| Hordeum vulgare | *HvNAS4* | AB011266 | BAA74583.1 | LL |
| Hordeum vulgare | *HVNAS5-1* | AB011267 | BAA74584.1 | LL |
| Hordeum vulgare | *HVNAS5-2* | AB011268 | BAA74585.1 | LL |
| Hordeum vulgare | *NASHOR1a* | AF136941 | AAD32650.1 | LL |
| Hordeum vulgare | *HvNAS7* | AB019525 | BAA74587.1 | LL |
| Hordeum vulgare | *NASHOR1b* | AB011269 | BAA74586.1 | LL |
| Hordeum vulgare | *NASHOR2* | AF136942 | AAD32651.1 | LL |
| Zea mays | *ZmNAS1;1* | GRMZM2G385200 | NP_001105504.2 | LL |
| Zea mays | *ZmNAS2;1* | GRMZM2G030036 | NP_001104862.2 | LL |
| Zea mays | *ZmNAS3* | GRMZM2G478568 | ONM07721.1 | LL |
| Zea mays | *ZmNAS2;2* | GRMZM2G124785 | ONL97243.1 | LL |
| Zea mays | *ZmNAS4* | GRMZM2G439195 | NP_001308337.1 | LL |
| Zea mays | *ZmNAS5* | GRMZM2G050108 | NP_001151345.2 | LL |
| Zea mays | *ZmNAS6;1* | GRMZM2G704488 | NP_001130727.1 | LL |
| Zea mays | *ZmNAS6;2* | AC233955.1_FGT003 | NP_001339057.1 | LL |
| Zea mays | *ZmNAS1;2* | GRMZM2G312481 | Zm00001d047656 | LL |
| Triticum aestivum | *TaNAS1-A* | KU529948 | AND77076.1 | LL |
| Triticum aestivum | *TaNAS1-B* | KU529951 | AND77079.1 | LL |
| Triticum aestivum | *TaNAS2-A* | KU529961 | AND77089.1 | LL |
| Triticum aestivum | *TaNAS2-D1* | KU529963 | AND77091.1 | LL |
| Triticum aestivum | *TaNAS2-D2* | KU529964 | AND77092.1 | LL |
| Triticum aestivum | *TaNAS3-A* | KU529947 | AND77075.1 | LL |
| Triticum aestivum | *TaNAS3-B* | KU529950 | AND77078.1 | LL |
| Triticum aestivum | *TaNAS4-A* | KU529958 | AND77086.1 | LL |
| Triticum aestivum | *TaNAS4-D* | KU529965 | AND77093.1 | LL |
| Triticum aestivum | *TaNAS4-U* | KU529966 | AND77094.1 | LL |
| Triticum aestivum | *TaNAS5-B* | KU529954 | AND77082.1 | LL |
| Triticum aestivum | *TaNAS6-A* | KU529955 | AND77083.1 | LL |
| Triticum aestivum | *TaNAS6-B* | KU529956 | AND77084.1 | LL |
| Triticum aestivum | *TaNAS6-D* | KU529957 | AND77085.1 | LL |
| Triticum aestivum | *TaNAS7-A1* | KU529960 | AND77088.1 | LL |
| Triticum aestivum | *TaNAS7-A2* | KU529959 | AND77087.1 | LL |
| Triticum aestivum | *TaNAS7-D* | KU529962 | AND77090.1 | LL |
| Triticum aestivum | *TaNAS9-A* | KU529949 | AND77077.1 | LL |
| Triticum aestivum | *TaNAS9-B* | KU529952 | AND77080.1 | LL |
| Triticum aestivum | *TaNAS9-D* | KU529953 | AND77081.1 | LL |
| Oryza sativa | *OsNAS1* | LOC_Os03g19427 | XP_015630629.1 | LL |
| Oryza sativa | *OsNAS2* | LOC_Os03g19420 | XP_015630628.1 | LL |
| Oryza sativa | *OsNAS3* | LOC_Os07g48980 | XP_015645101.1 | LL |
