# Supplementary material for: Investigation of Baseline Iron Levels in Australian Chickpea and Evaluation of a Transgenic Biofortification Approach
Source: Front Plant Sci. 2018 Jun 14;9:788. doi: 10.3389/fpls.2018.00788 (PMC6010650; doi:10.3389/fpls.2018.00788)
Supplement: Supplementary file 4 [file Table_4.DOCX]

Supplementary Material

Investigation of baseline iron levels in Australian chickpea and evaluation of a transgenic biofortification approach

Tan, Z.H.G.^1^, Das Bhowmik, S.S.^1^, Hoang, T.M.L.^1^, Karbaschi, M.R.^1^, Long, H.^1^, Cheng, A.^1^, Bonneau, J.P. ^2^, Beasley, J.T.^2^, Johnson, A.A.T.^2^, Williams, B.^1^, Mundree, S.G.^1^*

^1^Centre for Tropical Crops and Biocommodities, Queensland University of Technology, Queensland, Australia

^2^School of Biosciences, University of Melbourne, Victoria, Australia

*** Correspondence:** Prof Sagadevan Mundree: sagadevan.mundree@qut.edu.au

Supplementary Table 4. List of primers used in qPCR. The table provides the gene name, forward (Fw) and reverse (Rv) primers and PCR product length (bp).

| **Gene** | | **Sequences (5’-3’)** | **Expected amplicon size** |
| --- | --- | --- | --- |
| EF1α | Fw | TCCACCACTT GGTCGTTTTG | 64 |
|  | Rv | CTTAATGACA CCGACAGCAA CAG |  |
| GAPDH | Fw | CCAAGGTCAA GATCGGAATC A | 65 |
|  | Rv | CAAAGCCACT CTAGCAACCA AA |  |
| CaNAS2 | Fw | AGTAGTGCCT TTCTAAATGG CC | 116 |
|  | Rv | CATGTCACCA ATCCCCAACA T |  |
| CaNAS  (XP_004487761.1) | Fw | GTCACTCAAG TCTGATTCGA CC | 172 |
|  | Rv | TGAGGTGGTG CATGTTGTTA C |  |
| CaNAS  (XP_004488704.1) | Fw | TAGCAAGATC GTGGCATCGG | 135 |
|  | Rv | CCTCTACTCA TACCAACAAG TGC |  |
| CaNAS  (XP_004494544.1) | Fw | AGTGCTTTGT ATCTCATGGA GC | 133 |
|  | Rv | TGCATGCCCT TATATACGGC T |  |
