# Supplementary material for: Investigation of Baseline Iron Levels in Australian Chickpea and Evaluation of a Transgenic Biofortification Approach
Source: Front Plant Sci. 2018 Jun 14;9:788. doi: 10.3389/fpls.2018.00788 (PMC6010650; doi:10.3389/fpls.2018.00788)
Supplement: Supplementary file 5 [file Table_5.DOCX]

Supplementary Material

Investigation of baseline iron levels in Australian chickpea and evaluation of a transgenic biofortification approach

Tan, Z.H.G.^1^, Das Bhowmik, S.S.^1^, Hoang, T.M.L.^1^, Karbaschi, M.R.^1^, Long, H.^1^, Cheng, A.^1^, Bonneau, J.P. ^2^, Beasley, J.T.^2^, Johnson, A.A.T.^2^, Williams, B.^1^, Mundree, S.G.^1^*

^1^Centre for Tropical Crops and Biocommodities, Queensland University of Technology, Queensland, Australia

^2^School of Biosciences, University of Melbourne, Victoria, Australia

*** Correspondence:** Prof Sagadevan Mundree: sagadevan.mundree@qut.edu.au

Supplementary Table 5. List of primers used for cloning. The table provides the name of the gene, forward (Fw) and reverse (Rv) primers with underlined restriction sites and PCR product length (bp).

| **Gene** | | **Restriction site** | **Sequences (5’-3’)** | **Expected amplicon size (bp)** |
| --- | --- | --- | --- | --- |
| CaNAS2 | Fw | AscI | GGCGCGCCAT GGTTTGCAAG GAAGATATAT TAATC | 939 |
|  | Rv | Pac1 | TTAATTAATC ATTCTTCAAT GACCAATTCC TC |  |
| GmFER | Fw | SalI | GTCGACCCTA GGATGGCCCT TTCTTGCTCC | 793 |
|  | Rv | BstEII | GGTGACCTTA TACATGATCT TCATCGTGAA GAA |  |
