# Supplementary material for: Investigation of Baseline Iron Levels in Australian Chickpea and Evaluation of a Transgenic Biofortification Approach
Source: Front Plant Sci. 2018 Jun 14;9:788. doi: 10.3389/fpls.2018.00788 (PMC6010650; doi:10.3389/fpls.2018.00788)
Supplement: Supplementary file 6 [file Table_6.DOCX]

Supplementary Material

Investigation of baseline iron levels in Australian chickpea and evaluation of a transgenic biofortification approach

Tan, Z.H.G.^1^, Das Bhowmik, S.S.^1^, Hoang, T.M.L.^1^, Karbaschi, M.R.^1^, Long, H.^1^, Cheng, A.^1^, Bonneau, J.P. ^2^, Beasley, J.T.^2^, Johnson, A.A.T.^2^, Williams, B.^1^, Mundree, S.G.^1^*

^1^Centre for Tropical Crops and Biocommodities, Queensland University of Technology, Queensland, Australia

^2^School of Biosciences, University of Melbourne, Victoria, Australia

*** Correspondence:** Prof Sagadevan Mundree: sagadevan.mundree@qut.edu.au

Supplementary Table 6. List of primers used for PCR screening to detect the presence of the transgene. The table provides the name of the gene, forward (Fw) and reverse (Rv) primers and PCR product length (bp).

| **Gene** | | **Sequences (5’-3’)** | **Expected amplicon size (bp)** |
| --- | --- | --- | --- |
| CaNAS2 | Fw | GCATGTCACC AATCCCCAAC | 568 |
|  | Rv | CGCAGCATCA AAAGTGCTCC |  |
| GmFER | Fw | ATGGCCCTTT CTTGCTCCAA | 604 |
|  | Rv | GTTCTGCCAC ACTGTGAACG |  |
| Neomycin phosphotransferase II | Fw | ATTCGGCTAT GACTGGGCAC | 675 |
|  | Rv | TAAAGCACGA GGAAGCGGTC |  |
